# Arrhythmia Following Pulse Generator Change: What Is the Mechanism?

**DOI:** 10.19102/icrm.2022.13115

**Published:** 2022-11-15

**Authors:** Patrick M. Kozak, George M. Bodziock, Prashant D. Bhave

**Affiliations:** ^1^Section of Cardiovascular Medicine, Wake Forest University School of Medicine, Winston-Salem, NC, USA

**Keywords:** Electrocardiography, endless-loop arrhythmia, pacemaker complications

## Abstract

We present an unusual case of a “reverse” pacemaker-mediated endless loop arrhythmia with native atrioventricular conduction serving as the anterograde limb of the tachycardia circuit and the atrial depolarization stimulated by the pacemaker in response to the sensed ventricular QRS. The electrocardiogram findings can be explained by lead reversal in the header during pulse generator change, with the ventricular lead connected to the atrial port and the atrial lead connected to the ventricular port. Careful examination of the electrocardiogram with attention to the 2 closely spaced pacing stimuli separated by the paced atrioventricular delay provided an important clue for diagnosis this case.

## Case presentation

**Figure 1:**
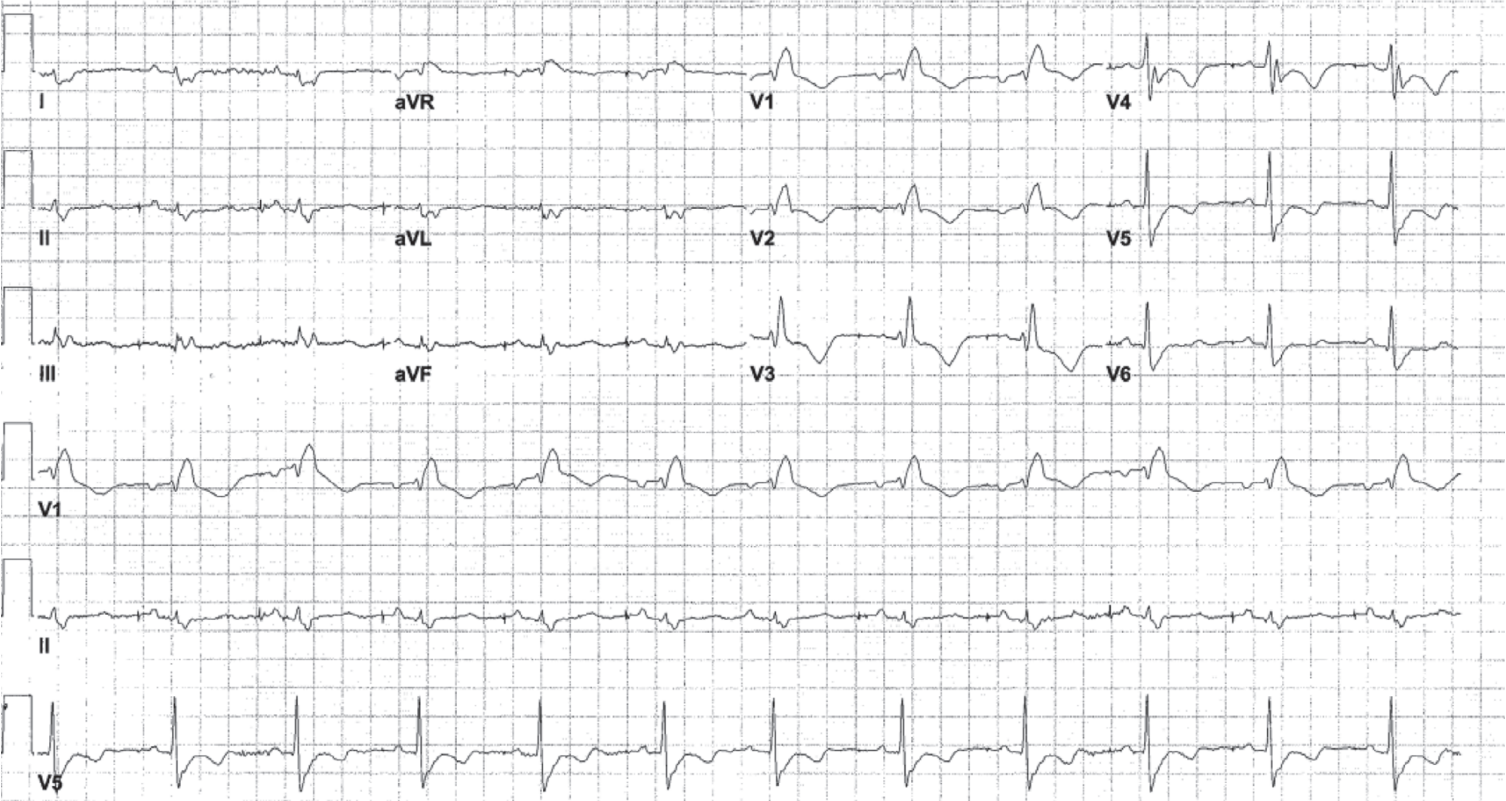
The baseline 12-lead electrocardiogram is shown. There is an atrial-paced rhythm with a long P–R interval and a right bundle branch block.

**Figure 2:**
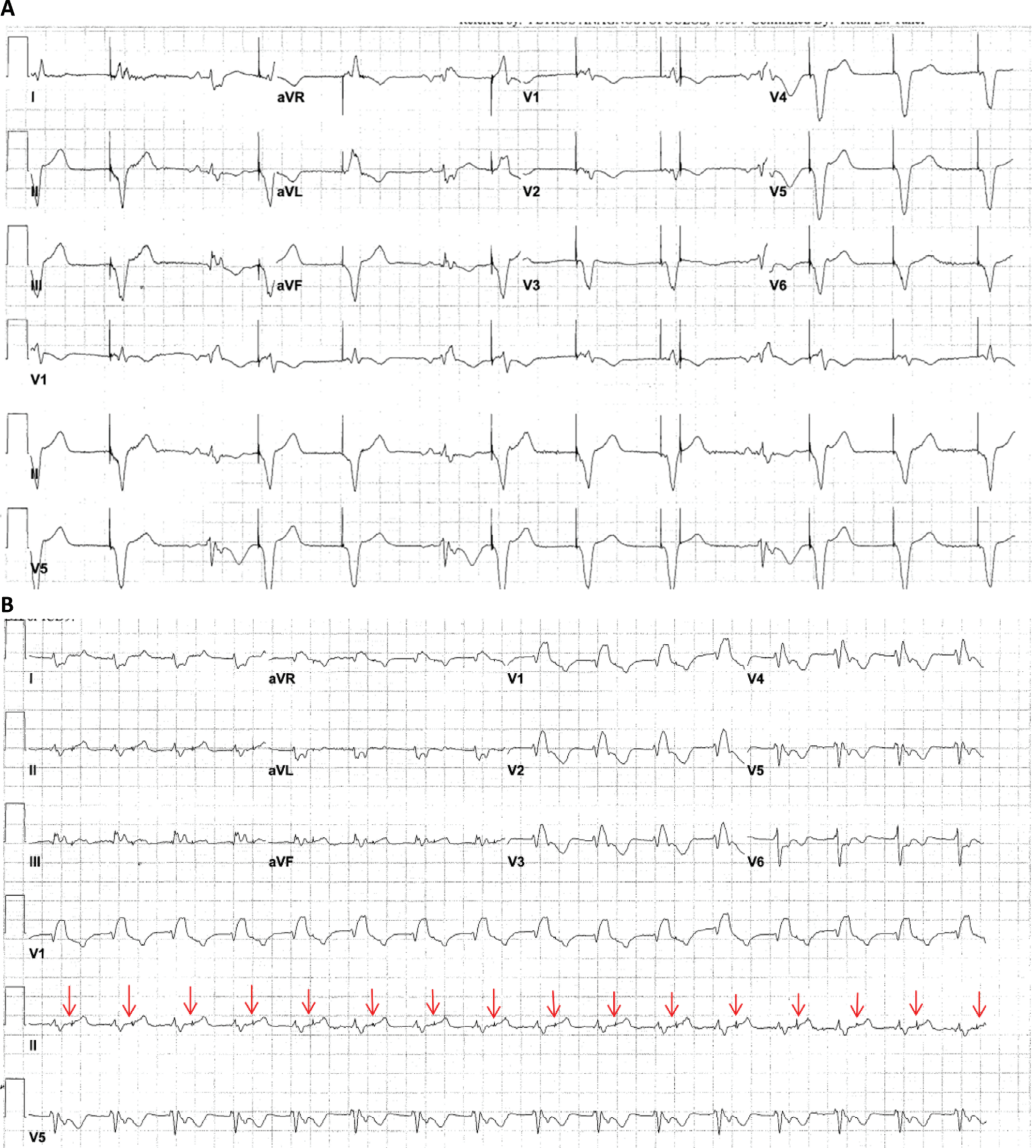
**A:** A 12-lead electrocardiogram shows intermittent ventricular pacing competing with sinus rhythm and native (atrioventricular) conduction. **B:** A wide QRS rhythm with a right bundle branch block morphology matching the patient’s native QRS is shown. There are pacing spikes in the T-wave after each QRS complex (red arrows).

A 32-year-old patient with congenital heart disease and a dual-chamber pacemaker that had reached its elective replacement interval was scheduled for a pulse generator change. The patient’s baseline 12-lead electrocardiogram (ECG) is shown in **Figure 1**. The pacing mode is DDD, and there is an atrial-paced rhythm with a conducted QRS complex with a right bundle branch block (RBBB) morphology. Following pulse generator change, the patient developed palpitations. The 12-lead ECGs in **Figures 2A and 2B** were taken after the procedure. We asked what might best explain the findings in **Figure 2A** and the rhythm in **Figure 2B**.

## Discussion

Pacemaker-mediated tachycardia (PMT) is a common phenomenon observed with dual-chamber pacing systems characterized by ventricular stimulation triggered by sensed atrial activity resulting from retrograde ventriculoatrial (VA) conduction. The native conduction system serves as the retrograde limb of the tachycardia circuit and the pacemaker serves as the anterograde limb. This circuit can generate an endless-loop arrhythmia. On the surface ECG, PMT classically manifests as a ventricular-paced rhythm at or below the programmed upper track rate of the device. We present a case of an unusual pacemaker-mediated endless-loop arrhythmia where careful examination of the ECG revealed the correct diagnosis.

**Figure 3:**
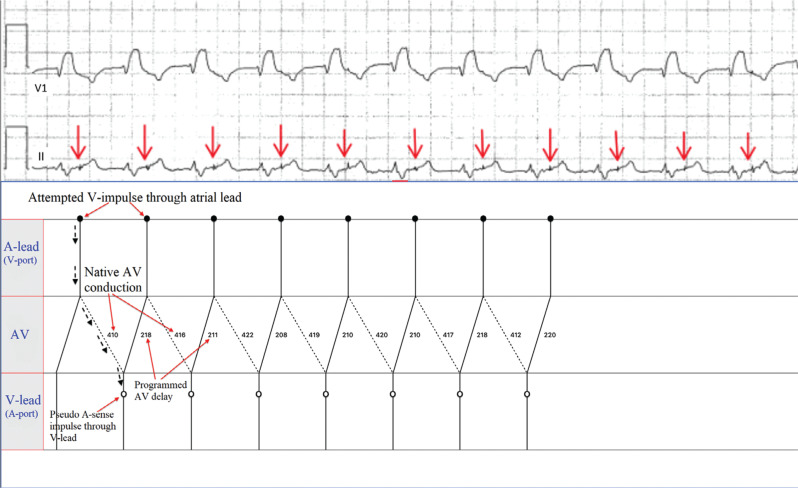
Ladder diagram of the endless-loop arrhythmia shown in **Figure 2B**. After the sensed atrioventricular delay timed out, this “pseudo-A–sensed event” triggered a sequential paced event, and the ventricular channel delivered a pacing stimulus to the atrium via the switched lead. This pacing stimulus generated an atrial depolarization that conducts anterogradely over the atrioventricular node, leading to a native QRS. The native QRS is sensed as an atrial event, and the cycle continues, leading to a “reverse” pacemaker-mediated endless-loop arrhythmia where native atrioventricular conduction serves as the anterograde limb of the circuit and the pacemaker serves as the retrograde limb of the circuit. *Abbreviation:* AV, atrioventricular.

The ECG findings in this case can be explained by the lead reversal in the header during pulse generator change, with the ventricular lead connected to the atrial port and the atrial lead connected to the ventricular port. **Figure 2A** shows intermittent ventricular pacing competing with native QRS complexes. The ninth QRS complex was captured by an initial pacing stimulus; however, a second pacing spike is present approximately 200 ms after the initial stimulus. The 2 pacing stimuli separated by the paced atrioventricular (AV) delay are key to the diagnosis in this case. Normally, the first pacing spike would generate an atrial-paced event; however, because of lead reversal in the header, it resulted in a ventricular-paced complex. After the programmed AV delay, the device then delivered a pacing stimulus through the ventricular channel if there was no “ventricular” sensed event. The paced QRS complexes that were not followed by the second pacing spike had subtle changes in T-wave morphology, suggesting retrograde VA conduction that inhibits pacing in the ventricular channel. **Figure 2B** shows a wide QRS rhythm at 96 bpm with an RBBB morphology suggestive of the patient’s native QRS. There were visible pacing spikes in the T-wave after each QRS complex. In **Figure 2B**, the pacemaker sensed the native QRS in the atrial channel (interpreting this as a P-wave). After the sensed AV delay timed out, this “pseudo-A–sensed event” triggered a sequential paced event, and the ventricular channel delivered a pacing stimulus to the atrium via the switched lead. This pacing stimulus generated an atrial depolarization that conducted anterogradely over the AV node, leading to a native QRS. The native QRS was sensed as an atrial event, and the cycle continues, leading to a “reverse” pacemaker-mediated endless-loop arrhythmia where native AV conduction serves as the anterograde limb of the circuit and the pacemaker serves as the retrograde limb of the circuit. **Figure 3** shows a ladder diagram of the proposed mechanism. The lead connections were revised, and thereafter the patient’s arrhythmia resolved.

Most cases of pacemaker-mediated endless-loop arrhythmia involve pacemaker stimulation of the ventricle; however, other mechanisms with pacemaker stimulation of the atrium have been described, either due to lead reversal at the header or double lead dislodgement.^1–4^ Careful examination of the ECG with attention to the 2 pacing stimuli separated by the paced AV delay provides an important clue for diagnosis in such cases. Preserved and prolonged AV conduction is necessary for sustaining this endless-loop arrhythmia. A short P–R interval may cause the R-wave to be within the post-ventricular atrial refractory period and break the circuit. The P–R interval in **Figure 2B** during the arrhythmia is longer than that on the preoperative ECG, likely due to a relative refractoriness of the antegrade conduction system created by a combination of a higher heart rate and the proximity of the paced p-wave to the prior conducted QRS complex. Although the original pacemaker telemetry tracings were not available to the authors, our proposed arrhythmia mechanism is the most likely explanation for the following reasons: (1) the fixed (separated by about 200 ms) relationship between the native QRS and the pacing spikes argues against under-sensing, cross-talk, and DDD programming with a long AV delay and (2) the temporal relationship between the pulse generator exchange and the arrhythmia suggests lead reversal at the header rather than double lead dislodgment, although chest radiographs should routinely be obtained if there is a concern for device-related arrhythmia. Careful attention must be paid to correct lead–header connections during device implant and pulse generator changes. The authors suggest a verbal “read back” of the last 3 digits of the lead serial number as each lead is connected to prevent this avoidable complication.

## Conclusion

We present an unusual case of a “reverse” pacemaker-mediated endless-loop arrhythmia with native AV conduction serving as the anterograde limb of the arrhythmia circuit and the atrial depolarization stimulated by the pacemaker in response to the sensed ventricular QRS.
